# Experimental and Theoretical Estimations of Atrazine’s Adsorption in Mangosteen-Peel-Derived Nanoporous Carbons

**DOI:** 10.3390/molecules28135268

**Published:** 2023-07-07

**Authors:** Juan Matos, Claudia P. Amézquita-Marroquín, Johan D. Lozano, Jhon Zapata-Rivera, Liliana Giraldo, Po S. Poon, Juan C. Moreno-Piraján

**Affiliations:** 1 Instituto de Ciencias Aplicadas, Facultad de Ingeniería, Universidad Autónoma de Chile, Santiago 8900000, Chile; 2 Escuela de Ingeniería de los Recursos Naturales y del Ambiente, Facultad de Ingeniería, Universidad del Valle, Calle 13 100-00, Cali 760035, Colombia; claudia.patricia.amezquita@gmail.com; 3 Departamento de Química, Facultad de Ciencias, Universidad de los Andes, Carrera Primera 18A-12, Bogotá 111711, Colombia; jd.lozanoc@uniandes.edu.co (J.D.L.); j.zapatar@uniandes.edu.co (J.Z.-R.); 4 Departamento de Química, Facultad de Ciencias, Universidad Nacional de Colombia, Carrera 45, Bogotá 111231, Colombia; lgiraldogu@unal.edu.co; 5 Unidad de Desarrollo Tecnológico (UDT), Universidad de Concepción, Barrio Universitario s/n, Concepción 4191996, Chile; ppoonngwork@gmail.com

**Keywords:** nanoporous carbons, atrazine removal, kinetics, isotherms, DFT estimations

## Abstract

Nanoporous carbons were prepared via chemical and physical activation from mangosteen-peel-derived chars. The removal of atrazine was studied due to the bifunctionality of the N groups. Pseudo-first-order, pseudo-second-order, and intraparticle pore diffusion kinetic models were analyzed. Adsorption isotherms were also analyzed according to the Langmuir and Freundlich models. The obtained results were compared against two commercially activated carbons with comparable surface chemistry and porosimetry. The highest uptake was found for carbons with higher content of basic surface groups. The role of the oxygen-containing groups in the removal of atrazine was estimated experimentally using the surface density. The results were compared with the adsorption energy of atrazine theoretically estimated on pristine and functionalized graphene with different oxygen groups using periodic DFT methods. The energy of adsorption followed the same trend observed experimentally, namely the more basic the pH, the more favored the adsorption of atrazine. Micropores played an important role in the uptake of atrazine at low concentrations, but the presence of mesoporous was also required to inhibit the pore mass diffusion limitations. The present work contributes to the understanding of the interactions between triazine-based pollutants and the surface functional groups on nanoporous carbons in the liquid–solid interface.

## 1. Introduction

The remarkable increase in emerging organic pollutants (EOPs) in surface and underground water sources is the consequence of different industrial activities. It has been reported that EOPs persist in drinking water even after being treated using conventional methods [1,2,3,4,5,6,7,8]. Well-known pesticides, herbicides, and fungicides are widely used in agriculture to prevent, combat, and destroy any pest. However, most of them generate negative impacts on the environment and health [1,2,3,8,9]. For instance, atrazine is one of the most widely used herbicides worldwide [10,11]. It is characterized by a triazine ring substituted with chlorine, ethylamine, and isopropylamine, which makes it recalcitrant to biological degradation in nature [12]. Atrazine and its degradation products are toxic and highly resistant; remain for many years in water, plants, and animals; and interfere with the life cycles of many species [13,14,15,16]. Animal studies have shown that atrazine causes neuroendocrine and reproductive problems and affects the development of pregnancy [17]. In rats and rabbits, the observed effects include the deterioration of neurological and reproductive systems and a decrease in the fetal body weight, and at concentrations as low as 0.1 μg·L^−1^, induced hermaphroditism in frogs [18]. Albuquerque et al. [19] concluded that atrazine has common toxic effects in aquatic species such as amphibians, fish, and crustaceans, among others. In humans, atrazine generates different health complications, ranging from irritations to probable alterations in the functions of some organs, reproduction problems, alterations in hormonal levels, premature births, birth defects, and low birth weight. It also affects the immune system and the endocrine system and causes highly strung sensations [10,20,21,22,23].

In the USA, Australia, and several European countries, atrazine has been prohibited or restricted due to its repercussions on the environment and health [11,15,24]. For instance, the Water Framework Directive [25] included atrazine as one of the 33 priority substances to be monitored in European waters. In addition, atrazine has been included in the list of prohibited pesticides for the year 2022, being prohibited in 44 countries, including 27 member countries of the European Union, the United States, Switzerland, Germany, and the United Kingdom, among others [26]. The tolerance limit of atrazine in water for human consumption is established in the United States at 3 μg·L^−1^ [11], while the World Health Organization establishes a limit of 2 μg·L^−1^ [27], and the European Community set the limit in 0.1 μg·L^−1^ for any individual pesticide and 0.5 μg·L^−1^ for the total pesticides used [25]. However, several works have reported atrazine concentrations in drinking water ranging from 0.02 to 1.9 μg·L^−1^ [28,29,30]. In rural zones in the Ebro River (Spain), atrazine concentrations of 12–170 μg·L^−1^ were found [31], while 2.4–8.2 μg·L^−1^ have been reported [32] in a coastal lagoon in the north of the Adriatic Sea. Latin American and Caribbean countries permit atrazine applications without any restriction. The permissible limit of atrazine in drinking water in Colombia is 0.1 μg·L^−1^ individually and 1 μg·L^−1^ for the total pesticides used [33]. However, according to the Central America and Caribbean regional report from the United Nations Environment Program, up to 2.9 μg·L^−1^ atrazine was found in groundwater with extensive sugarcane cultivation [34], while in Mexico, near an agricultural zone, concentrations of 4.6–15.0 μg·L^−1^ were found [35].

The aforementioned studies show that atrazine’s concentration exceeded the established limits, which reflects the need to develop an efficient technological solution for the removal of this pollutant [13,16]. In this sense, due to its high porosity and surface area, efficiency, simplicity of design, and low costs, adsorption by nanoporous carbon is one of the best technologies available for pesticide removal [1,36,37]. It is well known that the type of precursors and the parameters of activation influence porosimetry and surface chemistry, as they are responsible for the type of adsorption sites in porous carbons [38,39,40,41,42,43,44,45]. For instance, Dasgupta and coworkers [44] have reported that the surface chemistry of nanoporous carbons appears to be the most important parameter to control the interactions with polar molecules such as nitrobenzene. In an earlier study, our group [45] reported that nanoporous carbons with a high contribution of micropores are efficient to remove atrazine. However, the influence of the surface chemistry of carbons was not explored in that work.

The novelty of the present work is to verify the role of interfacial interactions between atrazine adsorption and porous carbons prepared from mangosteen-derived chars. Experimental studies for ATZ adsorption were performed, and different kinetics and equilibrium parameters were obtained and compared as a function of the textural and surface chemistry properties of carbons. Several theoretical estimations, including dipolar moments, adsorption energies, and density of states, were performed using the density functional theory for pristine and different oxygen-containing groups on graphene layers. These groups were selected from Boehm’s titrations performed on two porous carbons prepared using different methods from a mangosteen-peel-derived char. The results obtained from different kinetics/equilibrium studies of atrazine adsorption were compared against two commercially activated carbons.

## 2. Results and Discussion

### 2.1. N_2_ Adsorption–Desorption Isotherms

**Figure 1:**
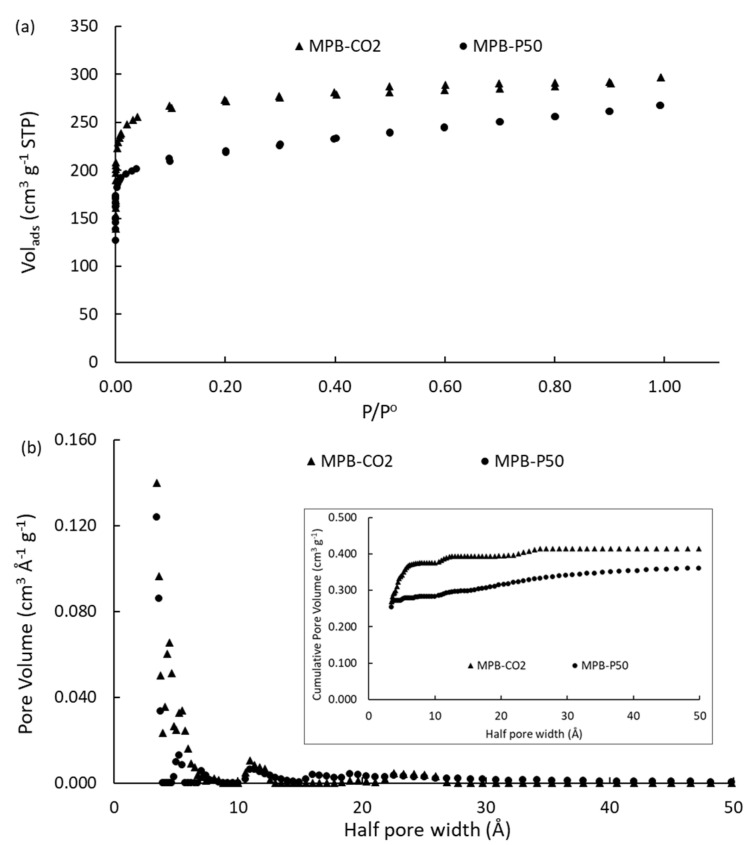
(**a**) N_2_ adsorption–desorption isotherms at −196 °C; (**b**) pore size distributions. The figure inset shows the cumulative pore volume on the activated carbons.

Figure 1a shows the N_2_ adsorption–desorption isotherms at −196 °C, and Figure 1b shows the pore size distributions of the two homemade carbons. The porous carbons presented type I(b) adsorption–desorption isotherm according to IUPAC classification [46,47], indicating that the pore size distributions (PSDs) were mainly composed of micropores [48], as can be seen in Figure 1b. The cumulative pore volume trend observed in the inset in Figure 1b suggests that the MPB-CO_2_ sample had a closed topology, with the main proportion comprising supermicropores (<1.0 nm). By contrast, the sample submitted to chemical activation (MPB-P50) mainly comprised large micropores (1.0–2.0 nm) and small mesopores (2.0–3.0 nm), even when the hysteresis loop was negligible. Table 1 shows a summary of the textural parameters and activation yields. For the sake of comparison, the commercially activated carbons [49] are also included.

The low yield observed in Table 1 for both MPB carbons suggests a high reactivity of the char during the activation [50,51]. For physical activation, direct gasification occurs under CO_2_ flow (pressure ca. 1 atm, flow ca. 100 mL·min^−1^) according to Equation (1).

C + CO_2_ → 2CO (1)

Chemical activation is an indirect gasification reaction via steam, as shown in Equation (2), which is formed from the thermal degradation of H_3_PO_4_, according to Equation (3).

C + H_2_O → CO + H_2_ (2)

2H_3_PO_4_ → P_2_O_5_ + 3H_2_O (3)

It is clear that S_BET_ and V_tot_ for the char (MPB) were negligible compared with the other carbons. A higher value of S_BET_ was observed for physical activation (ca. 1080 m^2^·g^−1^) than for chemical activation (ca. 847 m^2^·g^−1^), suggesting a more efficient interaction between the char and CO_2_, in agreement with a higher burn-off of ca. 76%. The present results are consistent with the experimental conditions used. In physical activation, 0.07 mols of CO_2_ flowed in 1 h activation at 800 °C, while only ca. 0.03 mols H_2_O were formed from H_3_PO_4_. Thus, keeping in mind that MPB char (for 100% C content) initially had ca. 0.083 mols, it is clear that physical activation should be more effective in the present conditions.

The PSD (Figure 1b) of the porous carbons is characterized by a large contribution to porosity in the range of 0.4 to 1.0 nm, with the highest contribution from ultramicropores, ranging from 0.4 to 0.7 nm for MPB-CO_2_. For MPB-P50, small mesopores had a low contribution in the range between 2 and 3 nm. The maximum contribution of micropore volume to the total volume of pores (V_mic_/V_tot_ = 0.92) was observed for the MPB-CO_2_ sample, with a mean pore width ca. 0.72 nm, which was ca. 52% lower than MPB-P50 (ca. 1.38 nm). It is interesting to highlight that commercially activated carbons were selected for the present study due to their similarities with the carbons prepared from mangosteen-peel-derived char. For instance, AC_M_ is mainly characterized by a micropore framework, with ca. 81% micropores and 0.96 nm of mean pore width, while AC_PC_ presents only 60% microporosity and a mean pore width of ca. 1.98 nm, which corresponds to the double of AC_M_.

### 2.2. Scanning Electron Microscopy (SEM) and Surface Analysis

**Figure 2:**
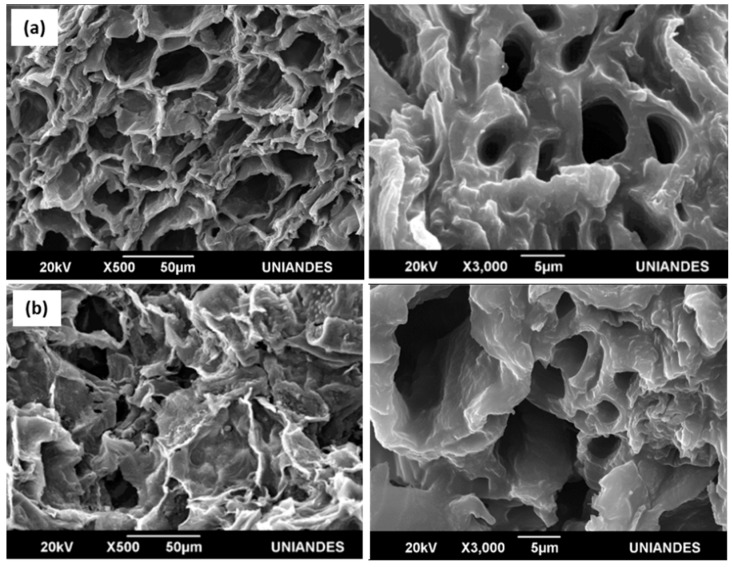
SEM images of the homemade carbons: (**a**) MPB-CO_2_; (**b**) MPB-P50.

Figure 2 shows SEM images of the activated carbons. It is clear the two materials are amorphous with an important roughness on the surface. Nevertheless, micro- or mesopores are not visible when using low-resolution SEM, thus indicating the formation of macropores in the two samples. Although SEM cannot be used to analyze porosity, it can be seen that the macropore framework in the MPB-CO_2_ sample (Figure 2a) seems to be more ordered along the surface in comparison with that observed for the MPB-P50 sample (Figure 2b). This observation could be associated with a lower reactivity in the chemical activation due to the low quantity of steam formed from the degradation of the activator.

**Figure 3:**
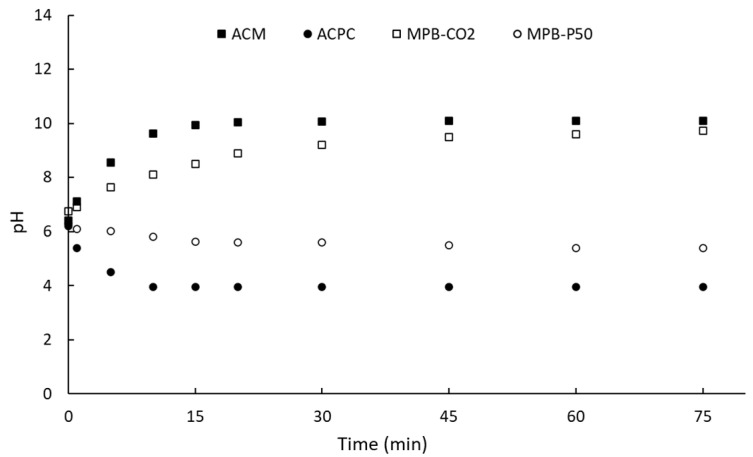
Evolution of pH of activated carbons as a function of contact time.

On the other hand, Figure 3 shows the evolution of pH as a function of time for an aqueous solution in contact with the activated carbons. The surface pH, also called the zero-point charge pH (pH_PZC_), of carbon materials can be estimated from the extrapolation of the plot at steady-state conditions [45]. For the sake of comparison, the two commercially activated carbons are also included in Figure 3.

After ca. 75 min, the steady-state condition was achieved. It is clear that commercially activated carbons and homemade nanoporous carbons had opposite surface pH. A surface pH of ca. 10.1, 3.9, 9.7, and 5.4 was observed for AC_M_, AC_PC_, MPB-CO_2_, and MPB-P50, respectively. This means that the surface pH of the activated carbons can be modulated as those of commercially activated carbons. Table 2 shows a summary of the surface pH (pH_PZC_) and the results obtained from Boehm’s titration. It is clear that the sample activated under CO_2_ flow had more lactone-like groups (0.532 mmol·g^−1^) than acidic groups, including a low proportion of carboxylic acids (0.053 mmol·g^−1^) and phenol (0.360 mmol·g^−1^).

Phenol is a weaker Brönsted acid than carboxylic acid [52], while lactone is a strong Lewis base. Therefore, it was expected to obtain a basic surface pH for MPB-CO_2_ in agreement with the surface pH obtained from the drift method (Figure 3). On the contrary, both carboxylic acids and phenolic groups were much higher for MPB-P50. It can be seen from Table 2 that MPB-P50 was characterized by a total acid group of ca. 1.163 mmol·g^−1^, which was ca. 2.8 times higher than MPB-CO_2_. This result agrees with the acid surface pH observed for MPB-P50 (Figure 3). Our group previously reported [44,49] the surface chemistry of the two commercially activated carbons.

AC_M_ was characterized by low phenolic groups and mainly lactone and pyrone groups in agreement with its basic surface pH. This means that both the surface pH (Table 2, pH_PZC_) and porosimetry properties (Table 1) of AC_M_ could be compared with those of MPB-CO_2_. By contrast, AC_PC_ was mainly characterized by an important proportion of carboxylic and phenolic groups, and accordingly, it had an acidic pH. MPB-P50 was also characterized by an acid surface comparable to that observed on AC_PC_. In addition, a primary proportion of the pore framework of MPB-P50 was composed of mesopores (Table 1), which allowed for a reasonable comparison with that observed for the commercial AC_PC_ (Table 1). 

### 2.3. Atrazine Adsorption

#### 2.3.1. Kinetic Studies

**Figure 4:**
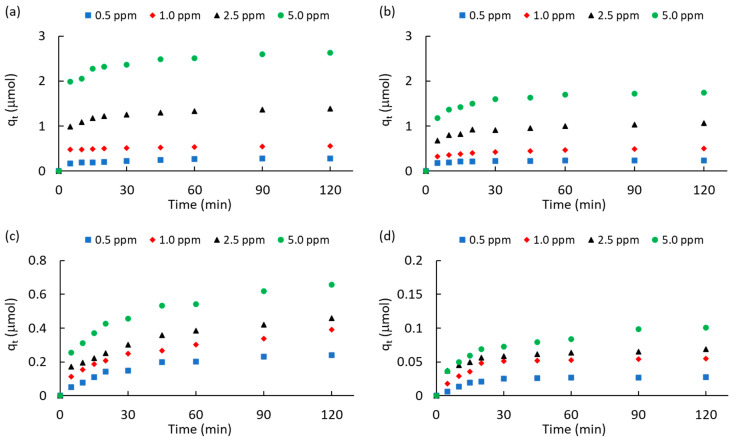
Kinetics of atrazine adsorption (q_t_) as a function of the initial concentration: (**a**) AC_M_; (**b**) AC_PC_; (**c**) MPB-CO_2_; (**d**) MPB-P50.

Figure 4 shows the kinetics of ATZ adsorption for different initial concentrations (0.5–5.0 ppm). Table 3 provides a summary of atrazine adsorbed at the equilibrium condition (after 120 min) and different kinetic parameters of adsorption. The two commercially activated carbons showed the highest ATZ uptake for all the initial concentrations. This result is attributed to a combination of a high surface area and a high total volume of pores (Table 1). However, although AC_PC_ was characterized by a higher surface area and total volume of pores than AC_M_ (Table 1), it is clear that AC_PC_ removed less ATZ (Table 3). For instance, AC_PC_ adsorbed ca. 15% and ca. 34% less ATZ than AC_M_ for 0.5 ppm and 5.0 ppm. These results suggest that the diffusion of ATZ molecules from the bulk of solution to the pores of adsorbents is more efficient for low concentrations of herbicide. However, this result seems to be contradictory with the dynamics of adsorption described using the intraparticle diffusion model (IPD) [53,54,55] since AC_PC_ had a higher number of mesopores than AC_M_ (Table 1). On the other hand, it cannot be overlooked that the acidic functional groups of AC_PC_ inhibited the diffusion of ATZ molecules to the pore framework. This inference seems to be reinforced by comparing the ATZ adsorbed on MPB-CO_2_ against MPB-P50. Although the surface area and total volume of pores of MPB-CO_2_ did not differ much from these values for MPB-P50, it is clear that atrazine adsorption was remarkably different. For instance, when increasing the initial concentration from 0.5 to 5.0 ppm, the ATZ adsorbed on MPB-CO_2_ was ca. 8.9, 7.1, 6.7, and 6.5 higher than that adsorbed on MPB-P50. This suggests that the acidic surface functional groups (mainly carboxylic acids and phenol) of MPB-P50 inhibited diffusion to the pore framework.

The molecular interactions associated with the mechanism of ATZ adsorption on the present porous carbons can also be interpreted in terms of the kinetic parameters of adsorption. Accordingly, the pseudo-first-order [53,56], pseudo-second-order [53,57], and intraparticle diffusion [53,54,55] models were analyzed. Table S1 (Supplementary Materials) shows a summary of the kinetic expressions and parameters obtained from the pseudo-first-order rate constant (k_1_), the pseudo-second-order rate constant (k_2_), the intraparticle (IPD) rate constant (k_p_), and the C constant attributed to the extension of the boundary layer thickness. Pseudo-first-order kinetics is associated with the reversible physisorption of molecules [58], while pseudo-second-order kinetics is associated with chemisorption phenomena [59], where strong interactions and bond formation may occur between the adsorbate and adsorbent. Figure S1 (Supplementary Materials) shows the plots for the atrazine adsorption on AC_M_ and MPB-CO_2_ at 0.5 and 5.0 ppm, respectively, in terms of the pseudo-first-order, pseudo-second-order, and intraparticle diffusion models. The regression values observed in Table 3 suggest that both AC_M_ and MPB-CO_2_ fitted very well with the pseudo-first-order and pseudo-second-order models, showing R^2^ > 0.95 in most cases. The average values for R^2^_k1_ and R^2^_k2_ were ca. 0.971 and 0.959 for AC_M_, while the values of 0.985 and 0.969 were estimated for MPB-CO_2_. Accordingly, it can be suggested that a mixture of physisorption and chemisorption mechanisms governs ATZ adsorption on carbons characterized by a basic surface and micropore framework. It is important to highlight that AC_M_ did not fit well with the intraparticle model, with an average R^2^_kp_ value of ca. 0.921, while a value of ca. 0.964 was obtained for MPB-CO_2_. It can be seen from Table 3 that at a low ATZ concentration (0.5 ppm), ATZ adsorbed at equilibrium conditions (q_eq_) was similar in both commercially activated carbons (0.282 μmol vs. 0.241 μmol).

By contrast, at high initial concentrations (5.0 ppm), q_eq_ was higher in AC_M_ than in AC_PC_ and ca. 4 times higher than in MPB-CO_2_ (2.632 μmol vs. 0.658 μmol). This result suggests that although the micropore contribution and surface pH of AC_M_ were almost similar to those of MPB-CO_2_, AC_M_ allowed for a better diffusion of molecules from the bulk of the solution to the pore framework. This ability was stronger at high initial concentrations. This inference is reinforced when the values of the C constant from the IPD model are compared between both carbons. Table 3 shows a monotonical increase in C values as a function of initial concentrations, from 0.146 to 1.921 μmols (13.2 times higher) for AC_M_, while for MPB-CO_2_, C values increased from 0.023 μmol to 0.189 μmol (8.2 times higher). In other words, high adsorption capacities for ATZ removal led to high values of the C constant. According to the IPD model, C was a measure of the boundary layer thickness of molecules approaching or in the vicinity of the adsorbent.

A similar analysis can be performed for AC_PC_ and MPB-P50. Figure S2 (Supplementary Materials) shows the plots for ATZ adsorption on AC_PC_ and MPB-P50 at 0.5 and 5.0 ppm, respectively. Table 3 shows that the linear regression factors for AC_PC_ fitted very well with the pseudo-first-order model (R^2^_k1_ of ca. 0.980). Conversely, this commercially activated carbon did not fit well with the pseudo-second-order model, showing an average R^2^_k1_ of ca. 0.927. In other words, even though the surface of AC_PC_ was acidic, ATZ was preferentially adsorbed via a physisorption mechanism, probably due to the high number of mesopores (Table 1). By contrast, ATZ was preferentially adsorbed via a chemisorption mechanism. This suggestion can be inferred from R^2^_k2_ values in Table 3, which are clearly higher than R^2^_k1_ values. At the same time, it can be seen from Table 3 that the C constants are clearly higher in AC_PC_ than in MPB-P50. For instance, C values increased from 0.181 μmol up to 1.181 μmol (6.5 times higher) in AC_PC_, while for MPB-P50, they only increased from 0.014 μmol up to 0.035 μmol when ATZ concentration increased from 0.5 up top 5.0 ppm.

Finally, with the exception of MPB-P50, k_1_ and k_2_ rate constants observed in MPB-CO_2_ and the commercially activated nanoporous carbons are in the same order of magnitude as values reported by Tan and coworkers [60] using corn-straw-derived porous carbons. In general, it is interesting to note that k_1_ and k_2_ values tended to decrease with an increase in concentration. This was particularly noticeable for k_2_ in most of the carbons studied in the present work. This result leads us to suggest that the chemisorption mechanism is favored at low concentrations, while at higher concentrations, physisorption and IPD model control the mechanism of adsorption. This result suggests that atrazine adsorption is highly dependent on the concentration of ATZ according to the intraparticle diffusion model [61]. In other words, at high concentrations, the energy required for the formation of bonds leading to chemisorption was higher since the number of surface interactions between ATZ molecules and the surface sites of adsorption decreased. These suggestions will be discussed in the following two sections using the equilibrium parameters obtained from the Langmuir and Freundlich isotherms as well as theoretical estimations.

#### 2.3.2. Adsorption Isotherms of Atrazine

**Figure 5:**
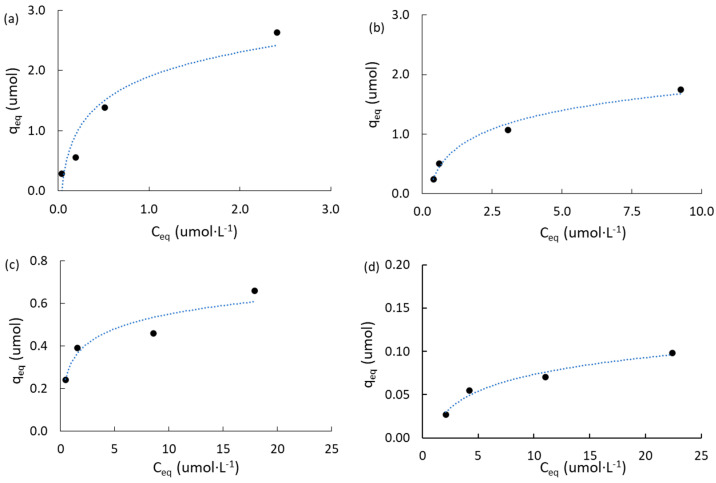
Adsorption isotherms of atrazine: (**a**) ACM; (**b**) ACPC; (**c**) MPB-CO_2_; (**d**) MPB-P50.

Table S2 (Supplementary Materials) shows a summary of the mathematical expressions used for equilibrium studies of atrazine adsorption according to the Langmuir model [62], and Freundlich model [63]. Figure 5 shows the adsorption isotherms obtained on commercially activated carbon and mangosteen-peel-derived carbons. The linear regression plots for both models are included in Figures S3 and S4 (Supplementary Materials). Table 4 is a summary of the equilibrium adsorption parameters obtained, including the maximum capacity for atrazine adsorption in the monolayer (q_m_, reported in μmol and mmol·g^−1^); the adsorption constant according to the Langmuir model (K_L_, reported in L·μmol^−1^); the adsorption constant according to the Freundlich model (K_F_, reported in mg·g^−1^ and mmol·g^−1^); and the Freundlich heterogeneity factor (n).

The linear regression factors according to the Freundlich model fit much better than those according to the Langmuir model for the commercially activated carbons (AC_M_ and AC_PC_). However, the opposite trend was observed in the mangosteen-derived carbons. Figure 5a shows that AC_M_ adsorbed more ATZ than AC_PC_ (Figure 5b) at initial concentrations higher than 1.0 ppm. The maximum capacity for ATZ adsorption in the monolayer for AC_PC_ was higher (0.466 mmol·g^−1^) than that obtained for AC_M_ (0.250 mmol·g^−1^). This result agrees with the higher specific surface area of AC_PC_ than that of AC_M_ (Table 1) and with a higher mesopore structure that led to the enhanced diffusion of ATZ molecules from the bulk of solution to the pore framework, as suggested by the lower values of the C constant from the IPD model in AC_PC_ than those in AC_M_ (Table 3) when ATZ was higher than 1 ppm. However, it can be hypothesized that in the present range of study (0.5–5.0 ppm), AC_M_ adsorbs more than one monolayer of atrazine molecules. This is inferred from the fact that the maximum capacity for ATZ adsorption in the monolayer (q_m_) according to the Langmuir model for AC_M_ was clearly lower (1.573 μmol, Table 4) than the value adsorbed at equilibrium (q_eq_) when the initial concentration of ATZ was 5.0 ppm (2.632 μmol, Table 3).

On the other hand, in the Freundlich isotherm, it is assumed that the surface of the adsorbent is energetically heterogeneous, where the adsorption sites have similar characteristic energies. It should also be considered that there were no lateral interactions between the adsorbed molecules, and therefore, only a monolayer was adsorbed. The heterogeneity factor of the Freundlich model (n_F_) was similar in both commercially activated carbons (1.79 and 1.72 for AC_M_ and AC_PC_), which suggests that only one monolayer should be adsorbed, which is contrary to the ATZ adsorption observed on AC_M_. In addition, it is clear from data in Table 4 that the adsorption constant according to the Langmuir model (K_L_) in AC_M_ was ca. 22 times higher than that observed in AC_PC_ (5.374 L·μmol^−1^ vs. 0.246 L·μmol^−1^). This result indicates that AC_M_ was characterized by a higher thermodynamic trend to adsorb ATZ than that observed on AC_PC_, even though the S_BET_ of the latter was higher. This trend is reinforced by the adsorption constant values obtained from the Freundlich model (K_F_), which were ca. 3 times higher in AC_M_ than in AC_PC_ (0.625 mmol·g^−1^ vs. 0.200 mmol·g^−1^). Accordingly, it can be suggested that the basic surface chemistry of AC_M_ could be responsible for significant electrostatic attraction among hydrated atrazine molecules, thus playing the main role in the adsorption of ATZ. 

Figure 5c,d show the results obtained for MPB-CO_2_ and MPB-P50, respectively, and the results obtained from the Langmuir and Freundlich models are summarized in Table 4. The linear regression plots for both models are included in Figure S4 (Supplementary Materials).

For instance, q_m_, K_L_, K_F_, and n_F_ parameters were ca. 4.0, 13.1, 9.6, and 2.1 times higher in MPB-CO_2_ than in MPB-P50. It is clear that MPB-CO_2_ had a higher capacity than MPB-P50 to adsorb atrazine, and this fact can be attributed to a higher BET surface area and a higher total volume of pores (Table 1). In addition, MPB-CO_2_ was characterized by a basic surface with a high surface pH compared with acidic groups and acid surface pH for MPB-P50 (10.1 vs. 3.9, Table 1).

It is interesting to highlight that the adsorption parameters observed in the mangosteen-derived carbons were remarkably lower than those observed in the commercially activated carbons. The low adsorption capacity observed for MPB carbons, mainly for MPB-P50 carbon, is attributed to the high proportion of acidic groups detected using Boehm titrations of carbons (Table 2). However, a more in-depth analysis using a specific surface technique such as X-ray photoelectronic spectroscopy (XPS) should be performed to complement this inference.

For MPB-CO_2_ carbon, this fact can be attributed to the high value of the heterogeneity factor according to the Freundlich model (n_F_), which mainly indicates that not only is the material characterized by different types of adsorption sites, but more importantly, it also has a high thermodynamic trend to adsorb ATZ. However, this was not the case for MPB-P50, with a value for n_F_ value of ca. 2.0, slightly higher than those observed in commercially activated carbons. Thus, it can be suggested that the high micropore proportion of the mangosteen-derived porous carbons, up to 92% and 78% for MPB-CO_2_ and MPB-P50, can be responsible for the low ATZ adsorption parameters. However, AC_M_ and MPB-P50 had comparable surface areas and pore frameworks (Table 1). In other words, it can be concluded that the thermodynamic trend to adsorb atrazine was favored by the presence of strong basic functional groups on the surface of the carbons. In addition, it should be highlighted that the average particle size of the mangosteen-derived carbons was ca. 350 μm, ca. 5 times higher than values observed for the commercially activated nanoporous carbons (ca. 75 μm). In a previous study [45], we have shown that the lower the size of particles, the higher the capacity of atrazine’s adsorption.

Finally, according to the Freundlich model, K_F_ values were ca. 134.9 mg·g^−1^, 43.2 mg·g^−1^, 15.3 mg·g^−1^, and 1.59 mg·g^−1^ for AC_M_, AC_PC_, MPB-CO_2_, and MPB-P50, respectively. Except for MPB-P50 carbon, these values are clearly higher than those reported by Tan and coworkers [60] for a porous carbon prepared from corn straw, with an adsorption capacity of ca. 4.6 mg·g^−1^. The loading used in the present work was ca. 0.05 g·L^−1^, which is similar to that reported by Tan and coworkers [60]. Thus, although the commercially activated carbons showed better capabilities to adsorb atrazine than the homemade MPB carbons, it should be noted that the mangosteen-derived porous carbon prepared via physical activation under CO_2_ flow (MPB-CO_2_) is a potential adsorbent, mainly due to its high BET surface area of ca. 1080 m^2^·g^−1^, compared with the value of 466 m^2^·g^−1^ reported for corn-straw-derived carbon [60]. In addition, the values obtained for the adsorption constant (Table 4) according to the Langmuir model (K_L_) were ca. 5.37 L·μmol^−1^, 0.25 L·μmol^−1^, 1.57 L·μmol^−1^, and 0.12 L·μmol^−1^, for AC_M_, AC_PC_, MPB-CO_2_, and MPB-P50, respectively. These values are remarkably higher than the value of ca. 0.009 L·μmol^−1^ reported for the corn straw-derived carbons [60] characterized by a high contribution of mesopores. Accordingly, the superior thermodynamic trend to adsorb ATZ, mainly for AC_M_ and MPB-CO_2_, can be attributed to the combination of their basic surface and the low number of mesopores (Table 1).

By contrast, in this study, the commercially activated and mangosteen-peel-derived nanoporous carbons showed lower q_m_ but higher K_L_ (except for MPB-P50) than the carbons prepared from hemp stem [42], with values of ca. 1.05 mmol·g^−1^ and ca. 0.14 L·μmol^−1^, respectively. The higher q_m_ reported for the hemp-stem-derived carbon can be attributed to a higher surface area (2135 m^2^·g^−1^) and to a much higher loading of adsorbent of ca. 3.0 g·L^−1^ (ca. 60 times higher) than that used in the present study. It should be highlighted that the K_L_ value obtained in MPB-CO_2_ porous carbons was ca. 11.2 times higher than that reported for hemp stem [42]. This comparison suggests that basic surface chemistry plays the most important role in ATZ adsorption, mainly at high concentrations. This suggestion is discussed in the following section using DFT estimations.

### 2.4. General Discussion and Theoretical Estimations

It is well known that the Langmuir model [62] considers all adsorption sites similar and finite. This model also assumes that interactions do not occur between adsorbed molecules. This means that the molecular density (*ρ*_surf_), also called surface density [64], can be estimated using Equation (4), where q_m_ is the maximum capacity of adsorption of atrazine obtained from Langmuir’s adsorption isotherms (Table 4), and S_BET_ is the specific surface area (Table 1).

*ρ*_surf_ = [(q_m_/S_BET_)·F] (4)

where F is a correction factor (F = 95.6) including Avogadro’s number, the weight of carbons (6.3 mg), and conversion factors to adjust the units of *ρ*_surf_ to adsorbed molecules·nm^−2^. The values estimated for the surface density of ATZ molecules adsorbed in the maximum capacity of adsorption (when q_eq_ = q_m_ = 1) were ca. 0.194, 0.226, 0.050, and 0.015 molecules·nm^−2^ for AC_M_, AC_PC_, MPB-CO_2_, and MPB-P50, respectively. Accordingly, the reciprocal of the surface density was the experimental value for the cross-sectional area (σ_ATZ_ = 1/*ρ*_surf_) of one atrazine molecule according to the Langmuir model. The values estimated for σ_ATZ_ were ca. 5.2, 4.4, 20.0, and 66.7 nm^2^·molecule^−1^ for AC_M_, AC_PC_, MPB-CO_2_, and MPB-P50, respectively. In other words, the higher q_m_ and the lower S_BET_, the higher the *ρ*_surf_ value and, accordingly, the lower the cross-sectional area of one atrazine molecule. These values of surface density suggest that the commercially activated carbons (AC_M_ and AC_PC_) were characterized by low repulsion forces among ATZ molecules, whereas mangosteen-peel-derived carbons (MPB-CO_2_ and MPB-P50) were characterized by high repulsion forces. For instance, AC_M_ adsorbed ca. 4 times more ATZ molecules than MPB-CO_2_. At the same time, AC_PC_ adsorbed ca. 15 times more ATZ molecules. This means that the electrostatic repulsion among atrazine molecules is the driving force for the adsorption of the pollutant. It is interesting to point out that the values obtained for σ_AT_ are much higher than that reported by Borisover and Graber [65], which was ca. 0.544 nm^2^ molecule^−1^, suggesting that more than one atrazine molecule is adsorbed in each adsorption site. The formation of atrazine’s molecular clusters adsorbed on the surface of porous carbons has been reported by our group [45].

In the lowest adsorption capacity observed in this work (q_eq_ values obtained from 0.5 ppm of ATZ, Table 3), the surface density values were ca. 0.035, 0.019, 0.021, and 0.003 molecules·nm^−2^ for AC_M_, AC_PC_, MPB-CO_2_, and MPB-P50, respectively. It can be seen that both AC_M_ and MPB-CO_2_ showed higher surface density than AC_PC_, suggesting that strong basic groups of carbons led to adsorption at a low initial concentration of ATZ; however, at high concentrations, both surface chemistry and porosimetry were responsible for the adsorption of ATZ.

**Figure 6:**
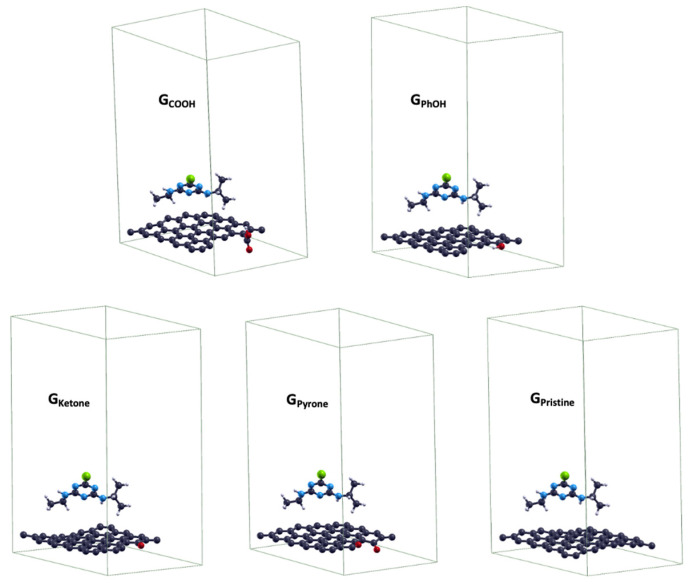
Optimized geometries of adsorbed systems in 1/1 monolayer using one atrazine molecule on a 5 × 5 graphene surface unit cell. O, N, Cl, and H atoms correspond to red, blue, green, and grey spheres, respectively.

According to this analysis, to verify the influence of surface functional groups on the nanoporous carbons in this study, the adsorption energy of atrazine (E_ads-ATZ_) was evaluated on one layer of pristine graphene (G_Pristine_) as well as after the introduction of different oxygen-containing functional groups. Periodic calculations were performed using the Perdew–Burke–Ernzerhof (PBE) exchange–correlation functional [66]. This functional has been proven to be reliable in the evaluation of adsorption energies of N- and Al-doped graphene [67] and carboxyl- and hydroxyl-decorated holes in graphene oxide [68]. Herein, theoretical calculations were limited to oxygen-functionalized graphene with pyrone (G_Pyrone_), ketone (G_Ketone_), phenol (G_PhOH_), and carboxylic acid (G_COOH_) groups. These groups were selected since they were identified from the Boehm titration study discussed above (Table 2). Figure 6 shows the optimized geometry for the atrazine adsorbed on the selected functionalized graphene.

The images in Figure 6 were generated on the basis of highest adsorbate coverage, corresponding to a surface coverage of 1/1 monolayer using one atrazine molecule on a 5 × 5 graphene surface unit cell. The adsorption energies of atrazine (E_ads-ATZ_) were calculated using Equation (5).

(E_ads-ATZ_) = [(E_Az-G_) − (E_G_ + E_Az_)] (5)

According to Equation (5), E_ads-ATZ_ can be estimated from the difference between the energy of the adsorbed system (E_Az-G_) containing both graphene and adsorbed atrazine and the sum of the energies of a clean graphene surface (E_G_) and an isolated atrazine molecule (E_Az_). Table 5 shows the theoretically predicted adsorption energies ranging from −0.169 eV for G_Pristine_ to −0.024 eV for G_PhOH_. The energy of adsorption is a thermodynamic potential that measures the spontaneous trend to adsorb molecules. Accordingly, it is clear that a higher and more negative E_ads-ATZ_ value resulted in more spontaneous atrazine adsorption. For instance, the lowest thermodynamic susceptibility to adsorb atrazine corresponded to the functionalization of graphene with acidic groups such as G_PhOH_ (−0.024 eV) and G_COOH_ (−0.048 eV). In contrast, the highest thermodynamic susceptibility corresponded to the functionalization of basic groups such as G_Ketone_ (−0.063 eV) and G_Pyrone_ (−0.099 eV). 

Pristine graphene showed the highest susceptibility to remove atrazine (−0.169 eV). This trend can be explained in terms of electron density; the polarization of the electronic density was smaller in G_Ketone_ and G_Pyrone_, and as expected, G_Pristine_ was the least polarized system. It is worth noting that the adsorption energies decreased with the dipole moment (µ) of clean graphene surfaces, except for G_PhOH_. In this system, the amplitude of µ was not as significant as in G_COOH_ due to the attenuation of the electronic delocalization in the whole layer, which was likely caused by the weak resonance of sp^3^ C atoms bonded to the O atom in the –OH group. Conversely, in G_COOH_, the attenuation was mostly caused by the orientation of the –COOH group with respect to the carbon surface, and hence the charge was polarized toward the –COOH moiety. In fact, G_COOH_ showed a high dipole moment. In summary, systems with larger electron delocalization led to large E_ads-ATZ_ values.

**Figure 7:**
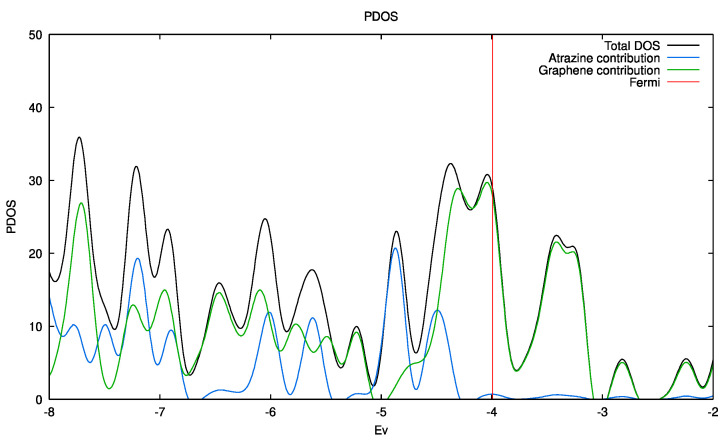
DOS (black line) and projected DOS on the pristine G_Pristine_ graphene (green line) and atrazine (blue line) with the PBE functional.

The density of states (DOS) and the projected density of states (PDOS) resulting from periodic calculations in the atrazine-adsorbed systems are presented in Figure 7. These calculations point to a conductor-like behavior for all systems, i.e., there was no bandgap. It is well known the lack of bandgap between the conduction and valence bands is associated with graphene, which has a continuous electronic density around the Fermi level. As can be seen in Figure S5 (Supplementary Materials), after adsorption, the materials remained almost unchanged in terms of their conductivity pattern independently of the type of oxygen-containing functional groups. 

Our equilibrium studies can be summarized as follows: The pore framework of the adsorbent played the most important role at low atrazine concentrations, with mesopores being the driving force behind the decrease in intraparticle pore diffusion limitations. Conversely, surface chemistry seemed to be the driving force for the adsorption of the herbicide at high concentrations of ATZ. It is concluded that the Langmuir and Freundlich models could be used to explain both the uptake and thermodynamic trends of atrazine adsorption on the current study’s commercially activated nanoporous carbons.

**Figure 8:**
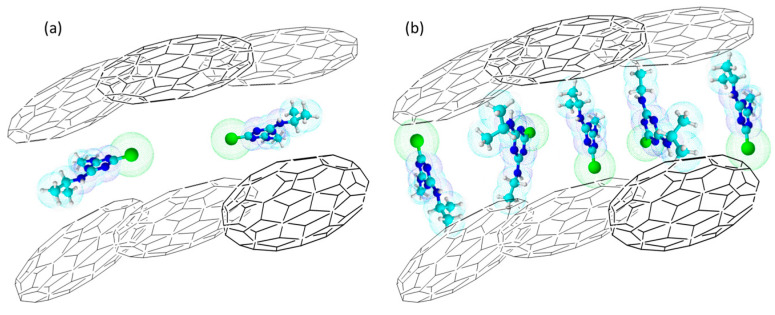
Schematic model for the atrazine adsorption on porous carbons: (**a**) low ATZ concentration. (**b**) high ATZ concentration.

Accordingly, Figure 8 shows a schematic model for the atrazine’s adsorption within slit-like pores of carbons considering low and high ATZ concentrations. Figure 8a shows the first case when atrazine was physically adsorbed in a parallel mode, forming a pseudo-layer within the carbon layers. In this case, the surface density was very low, with values of ca. 0.050 and 0.015 molecules·nm^−2^, but high cross-sectional areas of ca. 20.0 nm^2^·molecule^−1^ and 66.7 nm^2^·molecule^−1^ were observed in MPB-CO_2_ and MPB-P50, respectively. However, the present experimental values are remarkably higher than the theoretically calculated value of ca. 0.544 nm^2^ molecule^−1^ indicated by Borisover and Graber [65]. By contrast, at high ATZ concentrations (Figure 8b), the molecules were cumulated within the slit pores, and consequently, some of them were forced to rotate and adopt a vertical geometry mode, leading to high surface density values and low cross-sectional areas. In addition, this configuration led to high values of C constants (Table 3) according to the IPD model. This was specifically the case with AC_M_ and AC_PC_, with surface density values of ca. 0.194 and ca. 0.226 molecules·nm^−2^, respectively. Accordingly, the cross-sectional areas (σ_ATZ_ = 1/*ρ*_surf_) of the adsorbed atrazine obtained for the maximum coverage of adsorption were ca. 5.2 and ca. 4.4 nm^2^·molecule^−1^ for AC_M_ and AC_PC_, respectively. These values are almost one order of magnitude lower than those obtained in MPB-CO_2_ and MPB-P50 but still higher than the theoretical values reported [65], leading to the conclusion that more than one adsorption site was required for atrazine adsorption in the nanoporous carbons in this study. 

## 3. Experimental Procedures

### 3.1. Synthesis of Nanoporous Biochars

Mangosteen peel (*Garcinia mangosteen*), denoted as MP, was used as agricultural waste. A char sample was first prepared in a tubular furnace (Carbolite MFT, 12/38/400TM) via pyrolysis at 800 °C for 3 h under N_2_ flow (1 atm, 100 mL min^−1^) and denoted as MPB. Previous to pyrolysis, the peels were washed, dried, crushed, and sieved until achieving a particle size lower than 700 μm, with a mean particle size of ca. 350 μm. In the second step, two different nanoporous carbons were prepared from MPB using chemical and physical activation. Chemical activation was used by mixing 1 g of the char with 50 wt.% aqueous solution of H_3_PO_4_ with a 1:2 weight ratio for char:H_3_PO_4_. After observing a wetness impregnation condition (continuous stirring for ca. 1 h at 70 °C), the sample was activated at 800 °C for 1 h under N_2_ flow (ultra-high purity, 1 atm, 100 mL min^−1^). This sample was denoted as MPB-P50. A second nanoporous carbon was prepared at 800 °C via the physical activation of MPB under CO_2_ flow (ultra-high-purity, 1 atm, 1 h, 100 mL min^−1^) and denoted as MPB-CO_2_. For the sake of comparison, two different commercially activated carbons from Merck (ca. 90% microporous) and PureCarbon (ca. 60% microporous) were used and denoted as AC_M_ and AC_PC_, respectively. The average size of mangosteen-peel-derived carbons was ca. 5 times higher than the size of commercially activated carbons (ca. 75 μm).

### 3.2. Characterization

N_2_ adsorption–desorption isotherms were obtained at −196 °C in an Autosorb IQ2 equipment (Quantachrome). Samples were previously degassed at 250 °C for 6 h at high vacuum. The surface areas were estimated using the Brunauer–Emmett–Teller model (BET) using the multipoint N_2_ adsorption method [46,47], and the Dubinin–Astakhov (DA) method [48] was used to evaluate the micropore volume and pore size distribution (PSD).

The morphology of the samples was verified via scanning electron microscopy (SEM) using a JEOL microscope (6490-LV) operated at 20 kV. The functional surface groups of the carbons were quantified using the Boehm acid–base titration method [45,69]. In addition, the surface pH of carbons (pH_PZC_) was estimated using the drift pH method [37,70].

### 3.3. Kinetics and Equilibrium Studies of the Atrazine Adsorption

High-purity (99.9%, Riedel de Haen) atrazine (AT) was used. Table S3 (Supplementary Materials) summarizes some of the selected properties of AT, while Figure S6 shows its structural representation. The kinetics of adsorption were analyzed at a constant temperature of ca. 25 °C. In a typical test, 6.3 mg of carbon was suspended under constant stirring in 125 mL of ATZ solution with an initial concentration between 0.5 and 5.0 ppm (2.32–23.2 μmol·L^−1^; 0.29–2.9 μmol). The loading of adsorbent used in the present work was 0.05 g·L^−1^, ca. 20 times lower than reported in a previous study (1.0 g·L^−1^) [45]. This low loading decreases the costs associated with atrazine removal and prevents a high ATZ uptake, which can introduce inaccuracies in the estimations of the kinetic parameters of adsorption [53]. The time required to achieve the equilibrium of adsorption was determined from the kinetics of adsorption. Different kinetic parameters of adsorption were obtained from the pseudo-first-order [56], pseudo-second-order [57], and intraparticle diffusion models [54,55]. Table S1 (Supplementary Materials) provides a summary of the kinetic expressions used in the present study. Data of MB adsorbed at equilibrium conditions were normalized as a function of the sample’s weight. The amount of atrazine adsorbed q_ads-t_ (μmol) at time t was calculated using Equation (6), where C_o_ is the ATZ initial concentration (μmol·L^−1^), C_t_ is the concentration (μmol·L^−1^) at the time of adsorption t, and V is the volume of solution (0.125 L).

q_ads-t_ = (C_o_ − C_t_) × V (6)

The kinetics and equilibrium adsorption studies were performed without adding any buffer or electrolyte to control the pH. Several aliquots were taken off from the solution at different times and the concentration of ATZ in the solution was measured using UV–Visible spectroscopy in a Merck spectrophotometer set at 223 nm [30,42]. The results of atrazine adsorption isotherms were interpreted using the Langmuir [62] and Freundlich [63] equilibrium models. The equations used for the estimation of adsorption parameters are summarized in Table S2 (Supplementary Materials). The kinetics and equilibrium tests were conducted in duplicate, with a reproducibility better than 5%.

### 3.4. Theoretical Estimations

The adsorption energy of atrazine was evaluated based on a pristine graphene (G_Pristine_) structure. In order to verify the influence of the chemical surface of nanoporous carbons, computational estimations of the atrazine adsorption energy were also performed on graphene layers functionalized with oxygen-containing groups, including pyrone, ketone, phenol, and carboxylic acid groups, denoted as (G_Pyrone_), (G_Ketone_), (G_PhOH_), and (G_COOH_), respectively. In all cases, periodic DFT calculations were carried out using generalized gradient approximation (GGA), with the Perdew–Burke–Ernzerhof (PBE) exchange–correlation functional [66], as implemented in the Quantum Espresso package [71]. Ultrasoft pseudo-potentials available in the Quantum Espresso distribution repository were used in all calculations [72,73].

Graphene layers were optimized with a plane 5 × 5 hexagonal unit cell. The supercell parameters were a = 12.28 Å, b = 15.28 Å, c = 30 Å, α = ß = 90°, and γ = 120°. An additional 3.0 Å in b minimized the interlayer interactions and preserved the identity of the different substituents. Similarly, due to the 20 Å parameter in the c direction, the interaction between parallel layers could be ignored. In the geometry optimization calculations, valence electrons were described using plane waves with cutoff values of 150 Ry and 1500 Ry for energy and charge density, respectively. In such optimization calculations, a Γ-centered k-point Monkhorst–Pack sampling over the Brillouin zone, and the Gaussian broadening of 0.01 Ry as a smearing technique, were also used. These cutoffs were updated to 80 Ry and 800 Ry, respectively, with a 3 × 3 × 1 Γ-centered sampling over the Brillouin zone for the graphene layer and 3 × 3 × 3 Γ-centered for the adsorbed slabs. In the calculations of the projected density of states (PDOS), a denser k-point grid of 6 × 6 × 1 Γ-centered and 4 × 4 × 4 Γ-centered for graphene and adsorbed systems were used, respectively. The convergence thresholds for energy and forces were set up at 10^−4^ Ry and 10^−3^ Ry/Bohr for all calculations.

## 4. Conclusions

It is well known that activated carbons comprise extremely distorted defective graphene structures and not ideal graphene layers. The present work is scientifically important since the kinetic and equilibrium results presented here follow the same trend as those obtained through theoretical calculations. The characterization of the surface groups obtained from Boehm titration agrees with the preliminary results obtained from XPS, HRTEM, and EDS [45].

The present work contributes to the understanding of the interactions between triazine-based pollutants and the surface functional groups in nanoporous carbons in the liquid–solid interface. For instance, the kinetic models (pseudo-first-order, pseudo-second-order, and intraparticle diffusion models) and equilibrium parameters from the Langmuir and Freundlich models were correlated with the textural properties and surface chemistry of the nanoporous carbons.

The kinetic and equilibrium studies showed that at a low concentration, the pore framework played the most important role, where mesopores were the driving force inhibiting intraparticle pore diffusion limitations. This trend was the opposite at a high concentration of atrazine, where the surface chemistry seemed to be the driving force for the adsorption of the herbicide. The Langmuir and Freundlich models could be used to explain both the uptake and thermodynamic trends of atrazine adsorption in the commercially activated nanoporous carbons used in this study.

The results were compared against commercially activated carbons, and theoretical estimations were performed to verify the influence of different functional groups (acid and basic) on the thermodynamic trend to adsorb the pesticide. Accordingly, although the model used for the DFT estimations was based on a simplified notion (i.e., one that considers a nanoporous carbon to be constituted of graphene layers decorated with oxygen groups), the correlations found between the theoretical estimations of atrazine’s adsorption energy and the surface chemistry of the activated carbons are of major importance. The removal of atrazine expressed in terms of q_T_ was highly dependent on the surface area and the total pore volume, mainly, micropores. However, in terms of K_L_, the thermodynamic trend to adsorb atrazine increased with the increase in the surface pH of the adsorbent. This experimental fact was demonstrated with theoretical estimations of adsorption energy as a function of the polarization of the graphene layer in the presence of different functional groups.

In summary, the mechanism of ATZ adsorption seems to be a combination of physisorption and chemisorption, and both the surface chemistry and porous framework of carbons are the driving forces controlling the mechanism. A general conclusion drawn is that mangosteen peels can be potentially used as a biomass residue for the sustainable preparation of efficient adsorbent for the removal of pesticides such as atrazine, an important and dangerous problem in Latin American countries.

## Figures and Tables

**Table 1:** Summary of burn-off and textural properties of the mangosteen-derived (MPB-CO_2_ and MPB-P50) and commercially activated (AC_M_ and AC_PC_) carbons.

Samples	Yield ^a^ (%)	S_BET_^b^ (m^2^·g^−1^)	V_mic_^c^ (cm^3^·g^−1^)	W ^d^ (nm)	V_tot_^e^ (cm^3^·g^−1^)	V_mic_/V_tot_^f^ (%)
MPB	35	20	0.001	--	0.030	0.03
MPB-CO_2_	24	1080	0.420	0.72	0.459	0.92
MPB-P50	32	847	0322	1.38	0.414	0.78
AC_M_^g^	--	775	0.402	0.96	0.495	0.81
AC_PC_^g^	--	1240	0.390	1.98	0.650	0.60

^a^ Yields estimated from the initial and final weight (after activation). The pyrolysis yield is reported for MPB, while for the other samples, the final yield is the product of the two processes. ^b^ S_BET_ is the BET-specific surface area [46,47]. ^c^ V_mic_ is the volume of micropores according to the Dubinin–Astakhov model [48]. ^d^ W is the mean pore width according to the Dubinin–Astakhov model [48]. ^e^ V_Tot_ is the total volume of pores estimated at P/P_o_ ≈ 0.99. ^f^ V_mic_/V_tot_ is the micropore contribution to the pore framework. ^g^ Values are taken from reference [49].

**Table 2:** Summary of surface pH (pH_PZC_) and chemical functional groups of mangosteen-derived activated carbons evaluated using Boehm’s titrations.

Sample	Carboxylic	Lactones	Phenol	Total Acid	Total Basic	Total	pH_PZC_
	(mmol·g^−1^)	(mmol·g^−1^)	(mmol·g^−1^)	(mmol·g^−1^)	(mmol·g^−1^)	(mmol·g^−1^)	
MPB-CO_2_	0.053	0.532	0.360	0.413	0.532	0.945	9.7
MPB-P50	0.619	0.137	0.544	1.163	0.137	1.300	5.4

**Table 3:** Summary of kinetic parameters for the atrazine removal on porous carbons.

Carbon	ATZ (ppm)	q_eq_^a^ (μmol)	k_1_^b^ (min^−1^)	R^2^_k1_^c^	k_2_^d^ (μmol^−1^·min^−1^)	R^2^_k2_^e^	k_p_^f^ (μmol^−1^·min^−0.5^)	C ^g^ (μmol)	R^2^_kp_^h^
AC_M_	0.5	0.282	0.032	0.997	0.743	0.928	0.014	0.146	0.954
	1	0.556	0.024	0.996	0.638	0.966	0.009	0.458	0.981
	2.5	1.385	0.033	0.985	0.285	0.963	0.042	0.981	0.863
	5	2.632	0.021	0.906	0.130	0.979	0.073	1.921	0.889
AC_PC_	0.5	0.241	0.053	0.972	1.956	0.966	0.006	0.181	0.852
	1	0.503	0.028	0.996	0.394	0.950	0.020	0.299	0.955
	2.5	1.064	0.028	0.965	0.227	0.949	0.039	0.675	0.879
	5	1.742	0.041	0.985	0.349	0.846	0.060	1.181	0.844
MPB-CO_2_	0.5	0.241	0.033	0.977	0.378	0.944	0.022	0.023	0.931
	1	0.391	0.019	0.987	0.133	0.983	0.030	0.063	0.984
	2.5	0.459	0.024	0.996	0.185	0.971	0.035	0.099	0.977
	5	0.658	0.026	0.980	0.124	0.980	0.046	0.189	0.963
MPB-P50	0.5	0.027	0.052	0.919	18.796	0.911	0.002	0.014	0.698
	1	0.055	0.042	0.872	8.395	0.972	0.003	0.029	0.643
	2.5	0.069	0.023	0.900	2.684	0.993	0.003	0.041	0.875
	5	0.101	0.035	0.919	0.818	0.992	0.006	0.035	0.958

^a^ ATZ adsorbed after 120 min. ^b^ k_1_ is the pseudo-first-order rate constant. ^c^ R^2^_k1_ is the quadratic linear factor for k_1_. ^d^ k_2_ is the pseudo-second-order rate constant. ^e^ R^2^_k2_ is the quadratic linear factor for k_2_. ^f^ k_p_ is the intraparticle diffusion model (IPD) rate constant. ^g^ C is the boundary layer thickness constant for the IPD model. ^h^ R^2^_kp_ is the quadratic linear factor for the k_p_.

**Table 4:** Summary of the equilibrium parameters obtained for atrazine adsorption.

Carbon	q_m_^a^ (μmol)	q_m_ ^a^ (mmol·g^−1^)	K_L_^b^ (L·μmol^−1^)	R^2^_L_^c^	K_F_^d^ (mg·g^−1^)	K_F_^d^ (mmol·g^−1^)	n_F_ ^e^	R^2^_F_^f^
AC_M_	1.573	0.250	5.374	0.929	134.9	0.625	1.79	0.976
AC_PC_	2.937	0.466	0.246	0.934	43.2	0.200	1.72	0.942
MPB-CO_2_	0.565	0.090	1.574	0.952	15.3	0.071	4.12	0.923
MPB-P50	0.139	0.022	0.120	0.964	1.59	0.007	1.99	0.921

^a^ q_m_ is the maximum capacity for ATZ adsorption in the monolayer; ^b^ K_L_ is the adsorption constant according to the Langmuir model; ^c^ linear regression factor according to the Langmuir model; ^d^ K_F_ is the adsorption constant according to the Freundlich model; ^e^ n_F_ is the Freundlich heterogeneity factor; ^f^ linear regression factor according to the Freundlich model.

**Table 5:** Summary of adsorption energies of atrazine (E_ads-ATZ_) and dipolar moment (µ) obtained in pristine and oxygen-containing groups in graphene layers.

System	G_PhOH_	G_COOH_	G_Ketone_	G_Pyrone_	G_Pristine_
E_ads-ATZ_ (eV)	−0.024	−0.048	−0.063	−0.099	−0.169
µ (D)	1.103	3.290	1.917	1.791	0.001

## Data Availability

Data are available upon request.

## Supplementary Materials

The following supporting information can be downloaded at: https://www.mdpi.com/article/10.3390/molecules28135268/s1, Table S1. Kinetics model used for the analysis of ATZ adsorption. Table S2. Models for the adsorption’s isotherms and mathematical expression involved in the different equilibrium adsorption isotherms. Table S3. Atrazine’s selected properties. Figure S1. Kinetics treatments for ATZ adsorption on ACM (a–c,g–i) and MPB-CO_2_ (d–f,j–l). 0.5 ppm: (a–f); 5.0 ppm: (g–l). Pseudo first-order: (a,d,g,j); Pseudo second-order: (b,e,h,k); Intraparticle diffusion model: (c,f,i,l). Figure S2. Kinetics treatments for ATZ adsorption on ACPC (a–c,g–i) and MPB-P50 (d–f,j–l). 0.5 ppm: (a–f); 5.0 ppm: (g–l). Pseudo first-order: (a,d,g,j); Pseudo second-order: (b,e,h,k); Intraparticle diffusion model: (c,f,i,l). Figure S3. Linear regressions of Langmuir (a,c) and Freundlich (b,d) adsorption isotherms of atrazine. (a,b): ACM; (c,d): ACPC. Figure S4. Linear regressions of Langmuir (a,c) and Freundlich (b,d) adsorption isotherms of atrazine. (a,b): MPB-CO_2_; (c,d): MPB-P50. Figure S5. DOS (black line) and projected DOS on the oxygen-containing graphene (green line) and atrazine (blue line) with PBE exchange–correlation functional. Figure S6. Molecular structure of atrazine (C8H14ClN5).

## Sample Availability

Samples of the compounds are available upon request.
