# Supplementary material for: Experimental and Theoretical Estimations of Atrazine’s Adsorption in Mangosteen-Peel-Derived Nanoporous Carbons
Source: Molecules. 2023 Jul 7;28(13):5268. doi: 10.3390/molecules28135268 (PMC10343179; doi:10.3390/molecules28135268)
Supplement: Supplementary file 1 [file molecules-28-05268-s001.zip › molecules-2471068-supplementary.pdf]

Supplementary Material

Experimental and Theoretical Estimations of Atrazine's Adsorption in Mangosteen-Peel-Derived Nanoporous Carbons

Juan Matos [1,*], Claudia P. Amézquita-Marroquín [2,3], Johan D. Lozano [3], Jhon Zapata-Rivera [3], Liliana Giraldo [4], Po S. Poon [5] and Juan C. Moreno-Piraján [3,*]

[1] Instituto de Ciencias Aplicadas, Facultad de Ingeniería, Universidad Autónoma de Chile, Santiago 8900000, Chile
[2] Escuela de Ingeniería de los Recursos Naturales y del Ambiente, Facultad de Ingeniería, Universidad del Valle, Calle 13 100-00, Cali 760035, Colombia; claudia.patricia.amezquita@gmail.com
[3] Departamento de Química, Facultad de Ciencias, Universidad de los Andes, Carrera Primera 18A-12, Bogotá 111711, Colombia; jd.lozanoc@uniandes.edu.co (J.D.L.); j.zapatar@uniandes.edu.co (J.Z.-R.)
[4] Departamento de Química, Facultad de Ciencias, Universidad Nacional de Colombia, Carrera 45, Bogotá 111231, Colombia; lgiraldogu@unal.edu.co
[5] Unidad de Desarrollo Tecnológico (UDT), Universidad de Concepción, Barrio Universitario s/n, Concepción 4191996, Chile; ppoonngwork@gmail.com
* Correspondence: juan.matos@uautonoma.cl (J.M.); jumoreno@uniandes.edu.co (J.C.M.-P.)

Table S1. Kinetics model used for the analysis of ATZ adsorption.

Kinetic model	Equation	Reference
**Pseudo-first-order**	$\log(q_{eq} - q_t) = \log(q_t) - (k_1/2.303)\ t$	[53,56]
**Pseudo-second-order**	$(1/q_{eq} - q_t) = (1/q_{eq}) + k_2\ t$	[53,57]
**Intraparticle diffusion**	$q_t = C + k_p\ t^{1/2}$	[53-55]

The pseudo-first-order model showed in Eq. (1) where k_1 is the pseudo-first-order rate constant (min^{-1}) for the adsorption, q_t is the amount of MB adsorbed (in µmol), at time t (min) and q_{eq} is the amount adsorbed at equilibrium (in µmol).

$$dq_t/dt = k_1(q_{eq} - q_t) \qquad (1)$$

The integration of Eq. (1) at the initial conditions ($q_{at} = 0$ at $t = 0$) yields the Eq. (2):

$$\log(q_{eq} - q_t) = \log(q_{eq}) - (k_1/2.303).t \qquad (2)$$

In addition, a pseudo-second-order equation may be expressed by Eq. (3):

$$dq_t/dt = k_2(q_{eq} - q_t)^2 \qquad (3)$$

Where k_2 is the pseudo-second-order rate constant ($\mu mol^{-1}.min^{-1}$) for the adsorption. Applying the initial conditions, Eq. (3) can be integrated to obtain:

$$(1/q_{eq} - q_t) = (1/q_{eq}) + k_2.t \quad (4)$$

The influence of the intraparticle diffusion phenomena upon the adsorption capacity of MB was verified keeping in mind that the fractional approach to equilibrium change is done according to a function of $(D_t/r^2)^{1/2}$, where r is the radius of adsorbent particle and D is the effective diffusivity of solute within the particle. Thus, the initial rate according to the intraparticle diffusion model (IPD) is obtained from the liner regression of the curve $q_t = f(t^{1/2})$, expressed by the Eq. (5) where k_p is the IPD rate constant expressed in µmol $min^{-0.5}$, and C is the IPD capacity constant (µmol) attributed to the extension of the boundary layer thickness.

$$q_t = C + k_p.t^{1/2} \quad (5)$$

Table S2. Models for the adsorption´s isotherms and mathematical expression involved in the different equilibrium adsorption isotherms.

Model	Equation	Reference
Langmuir	$q_{eq} = q_m.K_L.C_{eq}/(1 + K_L.C_{eq})$<br>$\theta = q_{eq}/q_m$ = Surface coverage	[62]
Freundlich	$q_{eq} = K_F.C_{eq}^{1/n}$	[63]

q_{eq}: Amount of ATZ adsorbed at equilibrium (µmol); q_m: Maximum capacity for the ATZ adsorption in the monolayer (µmol; also expressed in mmol·g^{-1}); C_{eq}: Concentration in equilibrium (µmol·L^{-1}); K_L: adsorption constant according to Langmuir model (L·µmol^{-1}); K_F: adsorption constant according to Freundlich model (L); n: Freundlich´s heterogeneity factor.

Table S3. Atrazine´s selected properties.

Property	Property value
Molecular weight (g mol^{-1})	215.68
Vapour pressure (Pa)	3.8×10^{-5} at 25ºC<br>4.0×10^{-5} at 20ºC
Melting point (ºC)	175.8
Solubility in water (mg L^{-1})	28 at 20ºC<br>35 at 25ºC
Density (g cm^{-3})	1.187 at 20ºC
- Ln(pKa)	1.62 at 20ºC,<br>1.70 at 21ºC
Molecular size	Width: 0.96 nm<br>Depth: 0.84 nm<br>Cross sectional diameter: 0.544 nm^2

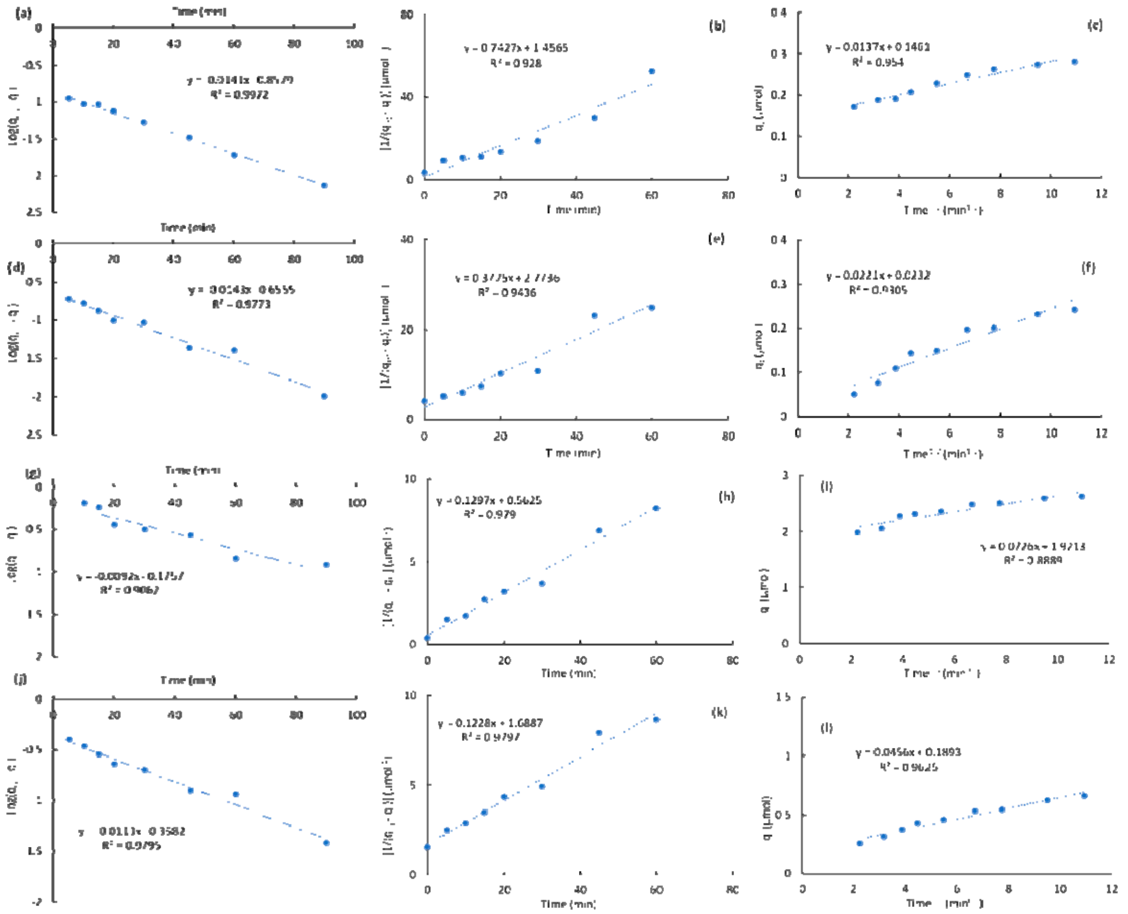

Figure S1. Kinetics treatments for ATZ adsorption on AC_M (a,b,c,g,h,i) and MPB-CO_2 (d,e,f,j,k,l). 0.5 ppm: (a,b,c,d,e,f); 5.0 ppm: (g,h,i,j,k,l). Pseudo first-order: (a,d,g,j); Pseudo second-order: (b,e,h,k); Intraparticle diffusion model: (c,f,i,l).

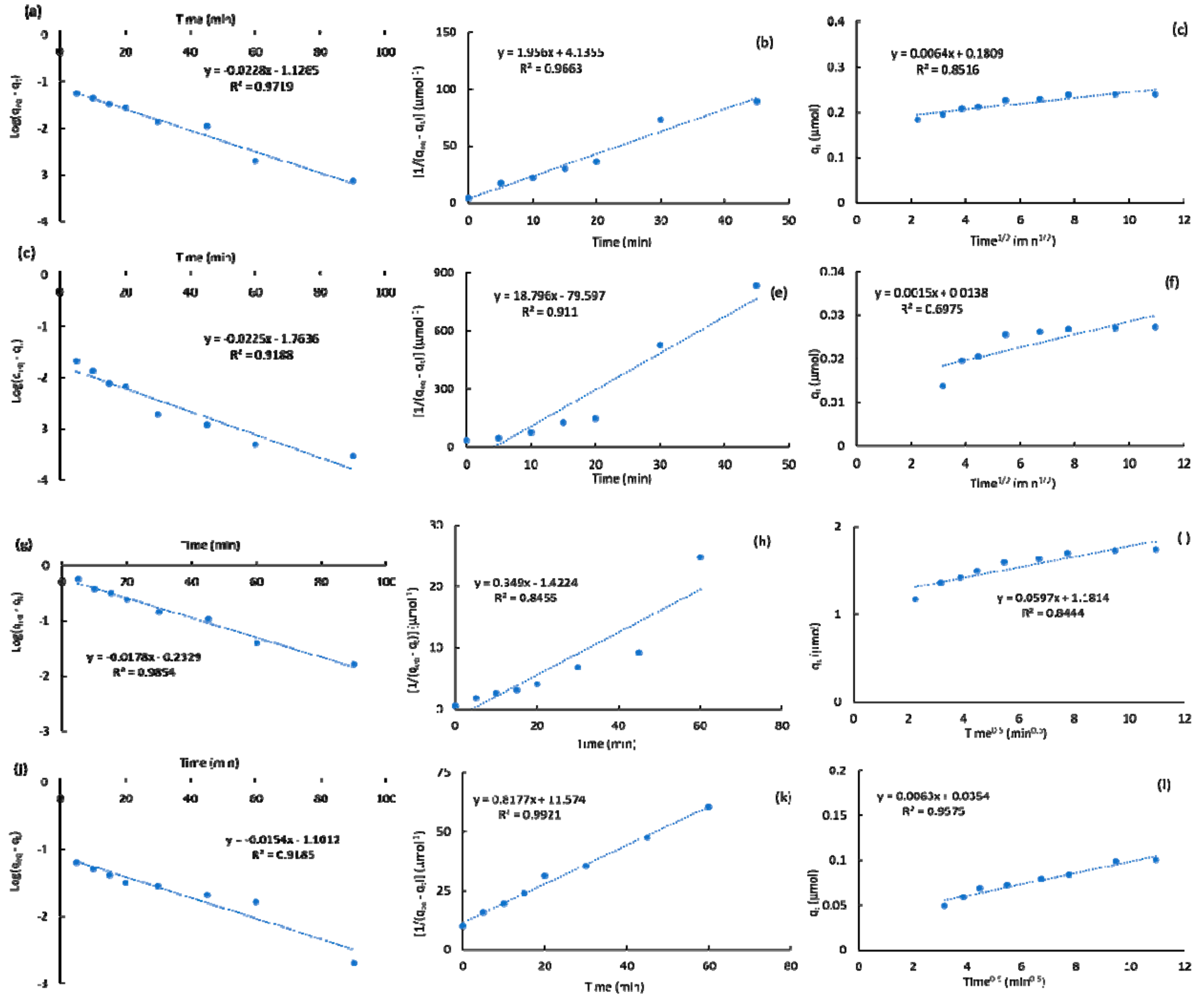

Figure S2. Kinetics treatments for ATZ adsorption on AC$_{PC}$ (a,b,c,g,h,i) and MPB-P50 (d,e,f,j,k,l). 0.5 ppm: (a,b,c,d,e,f); 5.0 ppm: (g,h,i,j,k,l). Pseudo first-order: (a,d,g,j); Pseudo second-order: (b,e,h,k); Intraparticle diffusion model: (c,f,i,l).

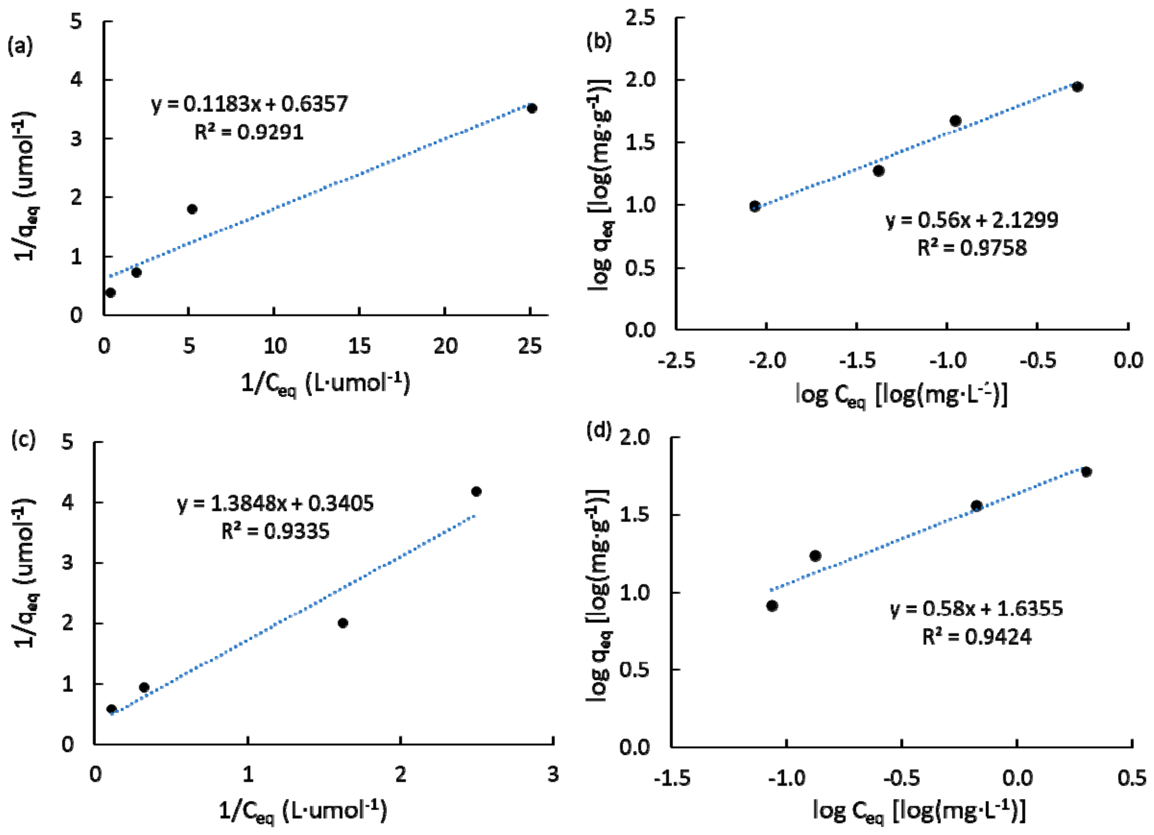

Figure S3. Linear regressions of Langmuir (a,c) and Freundlich (b,d) adsorption isotherms of atrazine. (a,b): AC_M; (c,d): AC_{PC}.

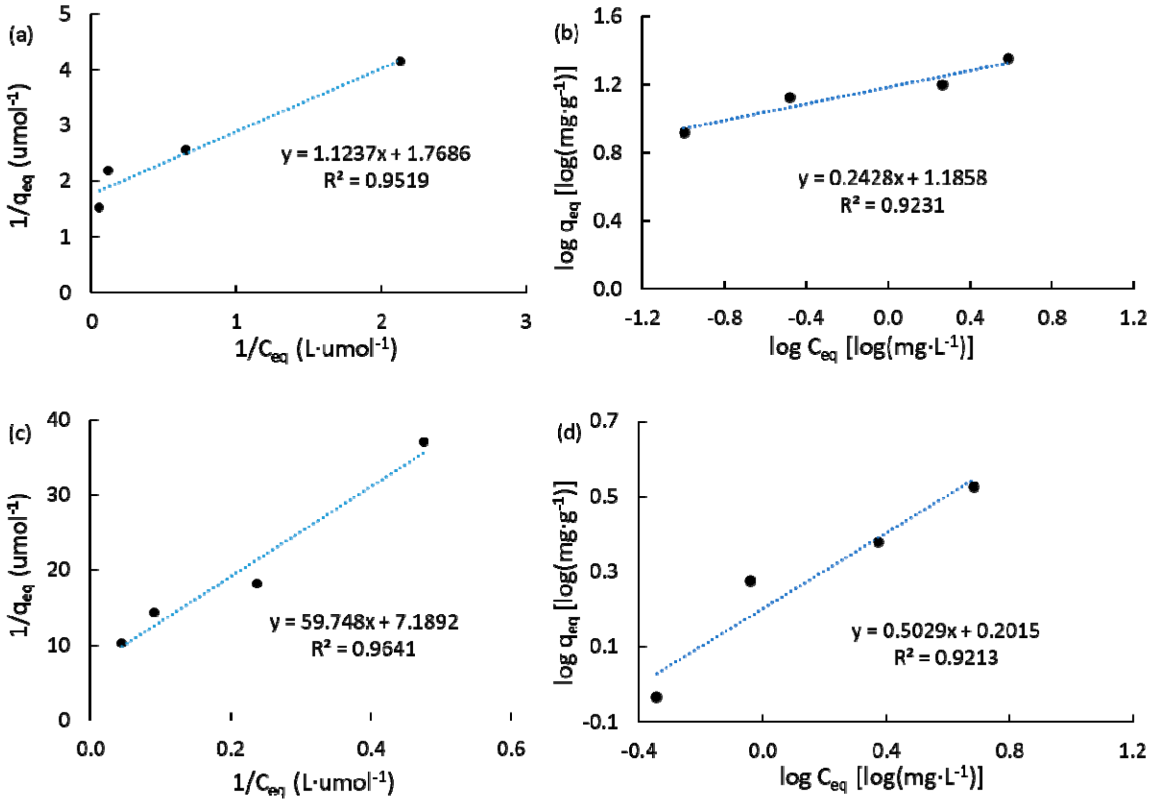

Figure S4. Linear regressions of Langmuir (a,c) and Freundlich (b,d) adsorption isotherms of atrazine. (a,b): MPB-CO_2; (c,d): MPB-P50.

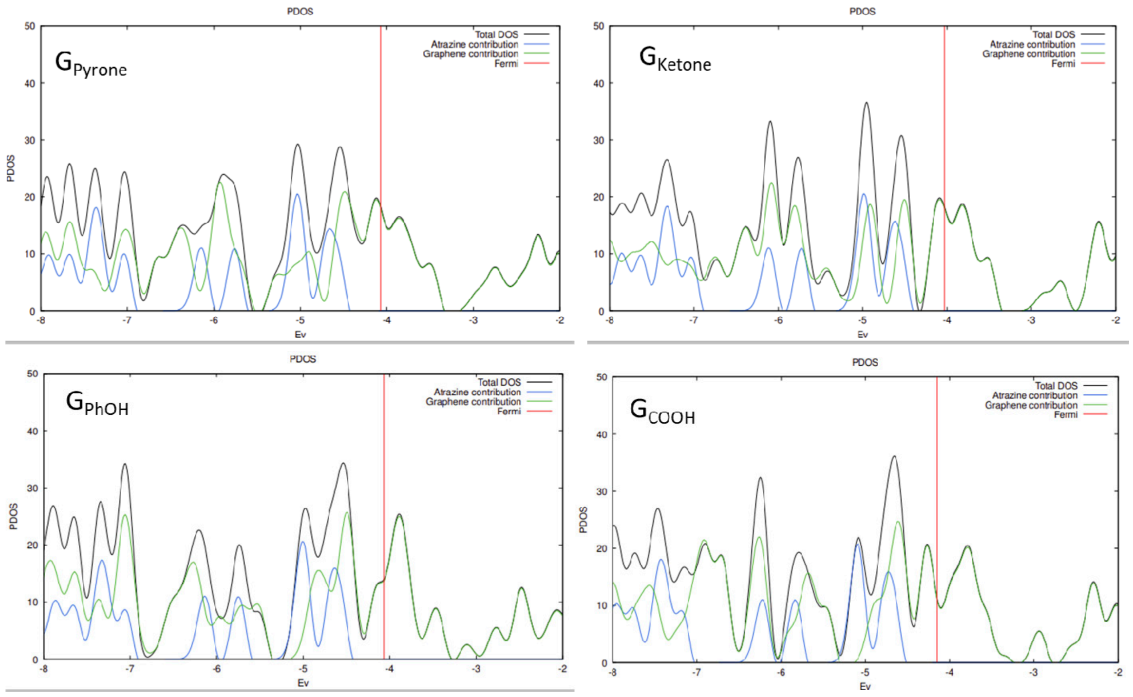

Figure S5. DOS (black line) and projected DOS on the oxygen-containing graphene (green line) and atrazine (blue line) with PBE exchange–correlation functional.

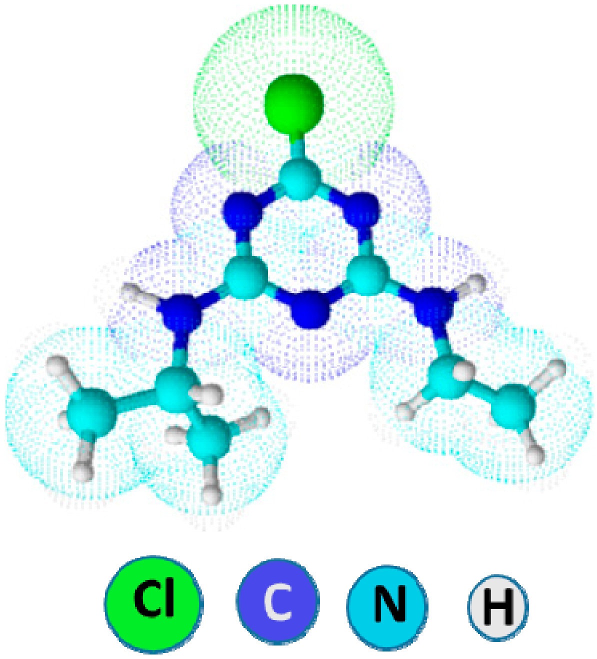

Figure S6. Molecular structure of atrazine ($C_8H_{14}ClN_5$).
